# Tau pathology associates with in vivo cortical thinning in Lewy body disorders

**DOI:** 10.1002/acn3.51183

**Published:** 2020-10-27

**Authors:** Nicola Spotorno, David G. Coughlin, Christopher A. Olm, David Wolk, Sanjeev N. Vaishnavi, Leslie M. Shaw, Nabila Dahodwala, James F. Morley, John E. Duda, Andres F. Deik, Meredith A. Spindler, Alice Chen‐Plotkin, Edward B. Lee, John Q. Trojanowski, Corey T. McMillan, Daniel Weintraub, Murray Grossman, David J. Irwin

**Affiliations:** ^1^ Penn Frontotemporal Degeneration Center Department of Neurology University of Pennsylvania Perelman School of Medicine Philadelphia PA USA; ^2^ Department of Neurology Perelman School of Medicine University of Pennsylvania Philadelphia Philadelphia PA USA; ^3^ Department of Neurosciences Health Sciences UC San Diego San Diego CA USA; ^4^ Department of Radiology Penn Image Computing and Science Laboratory University of Pennsylvania Perelman School of Medicine Philadelphia PA USA; ^5^ Alzheimer's Disease Center Department of Neuropathology Perelman School of Medicine University of Pennsylvania Philadelphia PA USA; ^6^ Department of Pathology and Laboratory Medicine Perelman School of Medicine University of Pennsylvania Philadelphia PA USA; ^7^ Parkinson's Disease Research Education and Clinical Center (PADRECC) Michael J. Crescenz Veterans Affairs Medical Center Philadelphia PA USA; ^8^ Center for Neurodegenerative Disease Research Perelman School of Medicine University of Pennsylvania Philadelphia PA USA; ^9^ Department of Psychiatry Perelman School of Medicine University of Pennsylvania Philadelphia PA USA; ^10^ Digital Neuropathology Laboratory Perelman School of Medicine University of Pennsylvania Philadelphia PA USA

## Abstract

**Objectives:**

To investigate the impact of Alzheimer’s disease (AD) co‐pathology on an in vivo structural measure of neurodegeneration in Lewy body disorders (LBD).

**Methods:**

We studied 72 LBD patients (Parkinson disease (PD) = 2, PD‐MCI = 25, PD with dementia = 10, dementia with Lewy bodies = 35) with either CSF analysis or neuropathological examination and structural MRI during life. The cohort was divided into those harboring significant AD co‐pathology, either at autopsy (intermediate/high AD neuropathologic change) or with CSF signature indicating AD co‐pathology (t‐tau/A*β*_1‐42_ > 0.3) (LBD+AD, *N* = 19), and those without AD co‐pathology (LBD−AD, *N* = 53). We also included a reference group of 25 patients with CSF biomarker‐confirmed amnestic AD. We investigated differences in MRI cortical thickness estimates between groups, and in the 21 autopsied LBD patients (LBD−AD = 14, LBD+AD = 7), directly tested the association between antemortem MRI and post‐mortem burdens of tau, A*β*, and alpha‐synuclein using digital histopathology in five representative neocortical regions.

**Results:**

The LBD+AD group was characterized by cortical thinning in anterior/medial and lateral temporal regions (*P* < 0.05 FWE‐corrected) relative to LBD−AD. In LBD+AD, cortical thinning was most pronounced in temporal neocortex, whereas the AD reference group showed atrophy that equally encompassed temporal, parietal and frontal neocortex. In autopsied LBD, we found an inverse correlation with cortical thickness and post‐mortem tau pathology, while cortical thickness was not significantly associated with A*β* or alpha‐synuclein pathology.

**Interpretation:**

LBD+AD is characterized by temporal neocortical thinning on MRI, and cortical thinning directly correlated with post‐mortem histopathologic burden of tau, suggesting that tau pathology influences the pattern of neurodegeneration in LBD.

## Introduction

Lewy body disorders (LBD) are pathologically characterized by abnormal accumulations of alpha‐synuclein (SYN) in Lewy bodies and Lewy neurites which are found in a clinically and pathologically heterogeneous group of neurodegenerative diseases, including Parkinson’s disease (PD), PD with dementia (PDD), and dementia with Lewy bodies (DLB).^1^ One potential factor contributing to such great clinical heterogeneity in LBD is the variable amounts of underlying AD co‐pathology found in LBD at autopsy^2^, ^3^, ^4^ AD neuropathology is common in LBD, with up to 50% LBD cases having sufficient plaque and tangle pathology at autopsy for a secondary diagnosis of intermediate‐to‐high AD.^5^ Clinically focused studies may be relatively insensitive to a meaningful signal from heterogeneous underlying biology in LBD.^4^, ^6^ Several studies have shown that coexistent AD pathology may have a significant impact on both the clinical presentation and the prognosis of LBDs.^7^ Indeed, efforts to stratify living cohorts based on biomarker patterns are informative for AD,^8^ but this approach is seldom used in LBD. Structural MRI may help to investigate in vivo the impact of AD co‐pathology in LBD. According to current clinical criteria, the qualitative absence of medial temporal lobe atrophy supports a diagnosis of DLB,^9^ and an AD‐like pattern of atrophy has been shown to predict cognitive decline even in non‐demented PD.^10^ Several studies also showed less severe cortical atrophy in DLB relative to Alzheimer’s disease (AD)^3^, ^4^, ^11^, ^12^; however prominent cortical atrophy does not exclude a diagnosis of DLB or PD. Comparisons between DLB and PDD have revealed substantial variability, with DLB generally reported to have greater cortical degeneration but inconsistent localization of atrophy.^13^, ^14^ Only rare in vivo studies have directly investigated the impact of AD co‐pathology on the extent of cortical atrophy in LBD,^4^, ^15^, ^16^ mostly using ordinal ratings of autopsy data for group‐wise comparisons. Recent advances in digital histopathology provide the ability to acquire more fine‐grained quantification of pathology necessary for detailed regional analyses.

In the present study, we examined differences in in vivo cortical thinning in LBD with either pathologically defined intermediate‐to‐high degrees of AD neuropathological change or CSF biomarker values consistent with AD co‐pathology (LBD+AD) compared to those without pathological or CSF evidence of significant AD co‐pathology (LBD−AD). We further compared cortical thinning in LBD groups to a set of 25 cases clinically diagnosed with amnestic AD. In the subset of LBD with autopsy tissue, we further tested the association of post‐mortem burden of tau, A*β* and SYN, as quantified by a highly sensitive, well‐validated digital histopathology method, to ante‐mortem regional cortical thinning. Based on previous neuropathological results^17^, ^18^ and recent molecular imaging data^19^, ^20^ showing a greater burden of tau pathology in the temporal lobe in LBD+AD compared to LBD−AD, we hypothesized that LBD+AD patients would have greater cortical thinning in temporal neocortex than LBD−AD, and that regional cortical thinning would correlate most closely with regional quantitative burden of tau pathology. Moreover, based on previous studies,^18^, ^19^ we hypothesized that regional neocortical thinning patterns in LBD would diverge from those classically associated with AD.

## Material and Methods

### Participants

Seventy‐two patients with a clinical diagnosis of LBD (PD: 2, PD‐MCI: 25, PDD: 10, DLB: 35)^9^, ^21^, ^22^ were included in the study. Participants were retrospectively selected from the Integrated Neurodegenerative Disease Database^23^ at the University of Pennsylvania. Only participants who underwent an MRI scan during life as well as either a cerebrospinal fluid (CSF) examination or a neuropathological evaluation were included in the study. *All patients included had Fazekas stage* ^24^ *≤1 vascular disease on clinical imaging*. The LBD cohort was divided into those cases with evidence of clinically significant AD pathology (LBD+AD, *N* = 19), either by neuropathological examination (intermediate‐to‐high AD neuropathologic change^25^, *N* = 7) or by previously established, pathologically validated CSF cut‐off^26^ (total tau/amyloid‐*β*_1‐42_ > 0.3; *N* = 12), and those with no or negligible AD co‐pathology (LBD−AD, no or low AD neuropathologic change at autopsy *n* = 14, CSF total tau/amyloid‐*β*_1‐42_ < 0.3; *N* = 39). A group of 25 patients with clinically defined amnestic AD^27^ and CSF total tau/ amyloid‐*β*_1‐42_ > 0.3 who matched the LBD+AD group for age, sex and MMSE score (see Table 1) and had comparable MRI scans (see below) was also selected. A group of 53 healthy participants with a Mini‐Mental State examination (MMSE) ≥27 and no evidence of a neurological condition, primary psychiatric disorder, or a medical condition causing cognitive difficulty was also included in the study as reference for the structural MRI comparison (see below). The research protocols were approved by the Institutional Review Board of the University of Pennsylvania, and all patients or caregivers acting on their behalf gave informed written consent in accordance with the Declaration of Helsinki. All patients were clinically diagnosed by experienced neurologists. Clinical and demographic information are reported in Table 1.

**Table 1:** Demographic and clinical information.

	LBD−AD	LBD+AD	AD	HC
Number (female)	53 (6)^1^, ^2^, ^3^	19 (7)	25 (11)	53 (20)
Clinical diagnosis	2 PD; 24 PD‐MCI; 8 PDD; 19 DLB	1 PD‐MCI; 2 PDD; 16 DLB	–	–
Age at MRI (std)	68.5 (7.4)	68.9 (5.0)	67.3 (6.3)	69.5 (6.2)
Education (std)	15.5 (2.3)	14.3 (2.9)^1^	15.9 (2.8)^4^	15.8 (2.2)
MMSE (std)	26.1 (4.2)^1^	20.1 (5.9)^1^, ^2^	20.0 (4.6)^1^, ^3^	29.2 (1.0)
Disease onset to MRI ‐ years (std.)	7.9 (6.0)^2^, ^3^	3.7 (2.7)	4.0 (3.1)	–
Neuropath. Cases	14	7	–	–

Data are reported as means. Abbreviations: PD, Parkinson’s disease; PD‐MCI, PD with mild cognitive impairment; PDD, Parkinson disease with dementia; DLB, Dementia with Lewy bodies; AD, Alzheimer’s disease; HC, healthy controls; MMSE, Mini‐Mental State Examination; std., standard deviation.

^1^ *P* < 0.05 between a patients group and the healthy controls.

^2^ *P* < 0.05 between LBD−AD and LBD+AD.

^3^ *P* < 0.05 between LBD−AD and AD.

^4^ *P* < 0.05 between LBD+AD and AD.

### Neuropsychological assessment

Global level of cognitive performance of the patients was assessed with the MMSE. For a subgroup of participants, the MMSE was not available while the Montreal Cognitive Assessment (MoCA) was. In these cases, the MoCA total score was converted to a MMSE score using published conversion criteria.^28^ Neither MMSE nor MoCA was available for four patients.

### Neuropathological examination

Neuropathological confirmation of patient diagnosis was obtained in 21 LBD participants (seven of these cases were included in a previous report of clinical‐pathological correlations^18^). Post‐mortem assessment was performed by experienced neuropathologists (EBL, JQT) using established criteria and immunochemistry.^18^, ^23^, ^29^ Two autopsy‐confirmed LBD cases presented with lateralized extrapyramidal symptoms with spatial difficulties and apraxia initially consistent with a clinical diagnosis of corticobasal syndrome but later in the disease developed more typical features of DLB, including well‐formed visual hallucinations, and thus met formal clinical criteria. LBD patients were grouped into those with an intermediate or a high level of AD neuropathologic change (ADNC) sufficient to contribute to AD (LBD+AD), and patients with no or low‐level AD pathology (LBD−AD)^18^ based on neuropathological criteria^25^ (see also Table 2).

**Table 2:** Information on the autopsy cohort.

	LBD−AD	LBD+AD
Number (female)	14 (0)	7 (1)
MRI‐death interval‐months (std)	21 (16)	21 (14)
Brain weight^1^	1346 (116)	1349 (115)
Braak stage^2^		
0	2	0
1	10	0
2	2	5
3	0	2
CERAD^2^		
0	9	0
1	2	1
2	2	2
3	1	4
Thal phase^2^		
0	9	0
1	0	0
2	3	1
3	2	6
AD copathology^2^		
None	9	NA
Low	5	NA
Intermediate	NA	5
High	NA	2
McKeith stage		
Brainstem	3	0
Limbic	4	1
Neocortical	7	6
Other copathology^2^, ^3^		
HS	0	1
LATE	1	2
AGD	1	0

LBD−AD, Lewy body disorders without evidence of clinically significant Alzheimer’s copathology; LBD+AD, Lewy body disorders with evidence of clinically significant Alzheimer’s copathology; AD, Alzheimer’s disease. AGD, argyrophilic grain disease; HS, hippocampal sclerosis; LATE, limbic‐predominant age‐related TDP‐43 encephalopathy; LBD, Lewy body disorder; NA, not applicable; SD, standard deviation.

^1^ Grams (std).

^2^ Number.

^3^ One LBD−AD patient had PSP co‐pathology largely confined to the brainstem and subcortical regions confirmed by Thioflavin S to differentiate AD tau neurofibrillary tauopathy from PSP tautopathy for Braak staging.

### Digital pathology

Five cortical regions were further examined using digital histologic methods: angular gyrus (ANG) Brodmann Area (BA) 39, primary visual cortex (VIS; BA17), anterior cingulate cortex (CING; BA24), superior temporal cortex (STC; BA22) and medial frontal cortex (MFC; BA 46). The anatomic location of these samples was guided by standard operating procedure that specifies the sampling at autopsy.^23^ Adjacent sections were immunostained in the Penn Digital Pathology Lab for phosphorylated tau (S202, T205; AT8;^30^ Thermo Scientific, Waltham, MA1:1K dilution without antigen retrieval), A*β* (NAB228;^31^ CNDR; 1:20K dilution with 88% formic acid antigen retrieval for 5 min), and phosphorylated alpha‐synuclein (phospho‐S129; MJF R‐13;^32^ Abcam, Cambridge, MA; 1:25 K dilution, Proteinase K (Fisher Scientific) pretreatment at 40°C for 15 min) utilizing an avidin–biotin complex detection system (VECTASTAIN ABC kit; Vector Laboratories) with 3,30‐diaminobenzidine as the chromogen as described^29^ for use in digital pathology experiments. Digital histopathological measures of each pathology was performed using Qu Path version 0.1.2 with validated methods of sampling and thresholding.^33^, ^34^ In brief, a belt‐transect method was used to randomly sample representative regions of cortex parallel to the cortical surface by manually segmenting the cortical ribbon and applying a random sampling of 175 μm^2^ tiles from 30% of the longest intact cortical ribbon. Color deconvolution intensity thresholds were optimized for each stain to detect and quantify the percent area occupied (%AO) for tau, A*β*, and SYN pathology and averaged across each random tile per slide, which was the main pathology outcome measure.^18^, ^29^ %AO values were validated by comparison with blinded ordinal ratings of pathology as previously reported.^29^ Missing or damaged tissues were excluded from analysis.

### Neuroimaging protocol

All the participants underwent a brain MRI scan on a Siemens 3T system (TIM Trio, Verio or Prisma; Siemens Healthcare, Erlangen, Germany). The scans took place between 2006 and 2019. T1‐weighted anatomical images were acquired on each participant, but four different protocols were employed over the many years of image acquisition. *Protocol a*: repetition time = 1620, echo time = 3.09, inversion time = 9500, voxel size = 0.98 × 0.98 × 1 mm, matrix = 192 × 256 × 160; *protocol b*: repetition time = 1800, echo time = 3.8, inversion time = 1080, voxel size = 1 × 1 × 1 mm, matrix = 256 × 256 × 160; *protocol c*: repetition time = 2300, echo time = 2.98, inversion time = 9000, voxel size = 1 × 1 × 1 mm, matrix = 240 × 256 × 208; *protocol d*: repetition time = 2300, echo time = 2.95, inversion time = 9000, voxel size = 1 × 1.02 × 1.02 mm, matrix = 192 × 236 × 197.

### Neuroimaging processing

#### Voxel‐wise analysis

Images were processed using *antsCorticalThickness.sh* implemented in Advanced Normalization Tools,^35^ which implements a symmetric diffeomorphic algorithm for registration to template^36^ with N4 bias‐field correction.^37^ Images were segmented into six classes using template‐based priors and estimates of cortical thickness (CT) were provided as voxel‐wise parametric maps. The CT maps were warped to the Montreal Neurological Institute (MNI) ICBM‐152 template^38^ using ANTs routines. CT maps were down sampled to a 2 mm^3^ resolution to minimize the processing load and smoothed using a Gaussian kernel with a standard deviation of 3 mm.

#### Regional analysis

The Lausanne2008 parcellation^39^ (scale 33) was warped to the individual T1‐weighted image using the *Easy‐Lausanne* pipeline (https://github.com/mattcieslak/easy_lausanne) ^40^ and ANTs routines and further masked by the CT map to assure only cortex was considered. Median CT values were extracted from each label. The regions of interest (ROIs) were further aggregated in seven macro‐ROIs for each hemisphere, namely lateral and medial parietal ROIs, lateral and ventral‐medial temporal ROIs, lateral and medial frontal ROIs and occipital ROI, which cover the entire neocortex (see Table 3).

**Table 3:** Definition of the macro ROIs.

macro‐ROI^1^	Label from the Lausanne 2008 parcellation (scale 33)^1^
Lateral parietal	Inferior parietal, superior parietal, supramarginal
Medial parietal	Isthmus cingulum, precuneus, posterior cingulate cortex
Lateral temporal	Inferior temporal, middle temporal, superior temporal, transverse temporal, banks of the superior temporal gyrus
Ventro‐medial temporal	Enterorhinal cortex, parahippocampus, fusiform
Lateral frontal	Caudate‐middle frontal, lateral orbitofrontal, pars opercularis pars orbitalis, pars triangularis, middle frontal, superior frontal
Medial frontal	Medial orbitofrontal, frontal pole, cuadal anterior cingulate cortex, rostral anterior cingulate cortex
Occipital	Lateral occipital, lingual

^1^ ROIs were combined hemisphere‐wise generating separate macro‐ROIs for each side.

### Statistical analysis

#### Demographic and clinical information

Statistical significance was tested with Mann‐Whitney U test and chi‐square test implemented in Python 3.7.

#### MRI voxel‐wise analysis

The voxel‐wise regression analysis was restricted to a gray matter mask based on ANTs tissue segmentation and group comparisons were performed with threshold‐free, cluster‐enhanced permutation statistics using FSL *randomise* (http://fsl.fmrib.ox.ac.uk/fsl/fslwiki/Randomise) with 10,000 permutations. Age, sex and imaging protocol were included as nuisance covariates in the model. Whole‐brain statistical significance was set at the family‐wise error (FWE) corrected threshold of *P* < 0.05.

### MRI regional analysis

To create a relative score comparable both within and across groups, the median CT values of each patient were transformed to w‐scores using the healthy participants as a reference group. W‐scores are analogous to z‐scores but they are adjusted for specific covariates,^41^ in the present study, age and sex. To compute a w‐score, multiple linear regressions were first performed in the control group between CT and age + sex. Then, the w‐score was computed by applying the following formula: [w‐score = ((patient’s CT)‐(value predicted by the model in the control group))/standard deviation of the residuals in control group]. Potential differences between comparable macro‐ROIs across the hemisphere were accessed with Student’s *t*‐test. Differences across groups were assessed using ANCOVA, including the imaging protocol as a nuisance covariate, and the Tukey’s post hoc test. Adjustment for multiple tests in the different regions were performed using Bonferroni correction. The analysis was implemented in Python 3.7.

### Association between cortical thinning and digital pathology

At the Penn FTDC, ROIs from the Lausanne2008 parcellation (scale 250) were combined in order to create new ROIs that more closely match the location of the tissue samples on which digital pathology was performed, based on a multidisciplinary comparison of MRI atlas and the gross anatomical atlas used for post‐mortem sampling. The new ROIs were warped to each T1‐weighted image and intersected with the CT map. The median CT value was then computed for each ROI and normalized with a w‐score procedure using the healthy participants as a reference group as described in the previous paragraph to account for systematic differences in CT across regions and to avoid bias in subsequent analyses. The %AO was log‐transformed with 0.001 imputed for regions with %AO of 0 based on established control signal which approximates 0.^29^ Values falling below the imputed value – three times the standard error of the mean were considered outliers and excluded. This led to the exclusion of a total of five data points (two from the tau dataset, 2%, and three from the A*β* dataset, 3%). Linear mixed effects models were fit for tau, A*β* and SYN including the ln of %AO, age at scan, sex, imaging protocol, the interaction between the ln‐%AO, interval between MRI and death, and the sampled ROIs as fixed variables, while a random factor was included for each participant to account for repeated pathology sampling in the same individual. CT was modeled as the dependent variable. The package *lme4* was used in Rstudio (v1.2.5001) to fit the mixed effects model, and a *P*‐value for the fixed effect of ln‐%AO was computed using the Kenward–Roger approach available in the package *pbkrtest*. Regional distribution of tau, A*β* and SYN were compared using multiple linear regression models including age at death and sex as nuisance covariates and chi‐square tests when appropriate.

## Results

### Demographic and clinical information

Our LBD cohort (PD/PDD/DLB) was stratified into biological groupings based on autopsy/CSF data to reflect those with (LBD+AD) and without (LBD−AD) clinically significant AD co‐pathology to test our hypotheses (Tables 1 and 2). We also included reference cohorts of healthy controls and amnestic AD patients with an AD CSF biomarker profile. The LBD−AD group had a significantly longer disease duration at the time of the MRI scan than both the LBD+AD and the AD groups (both *P* < 0.01) as well as a larger proportion of males (all *P* < 0.05). MMSE differed between healthy participants and all patient groups (all *P* < 0.001). See Table 1 for complete demographic information.

### Voxel‐wise analysis

**Figure 1:**
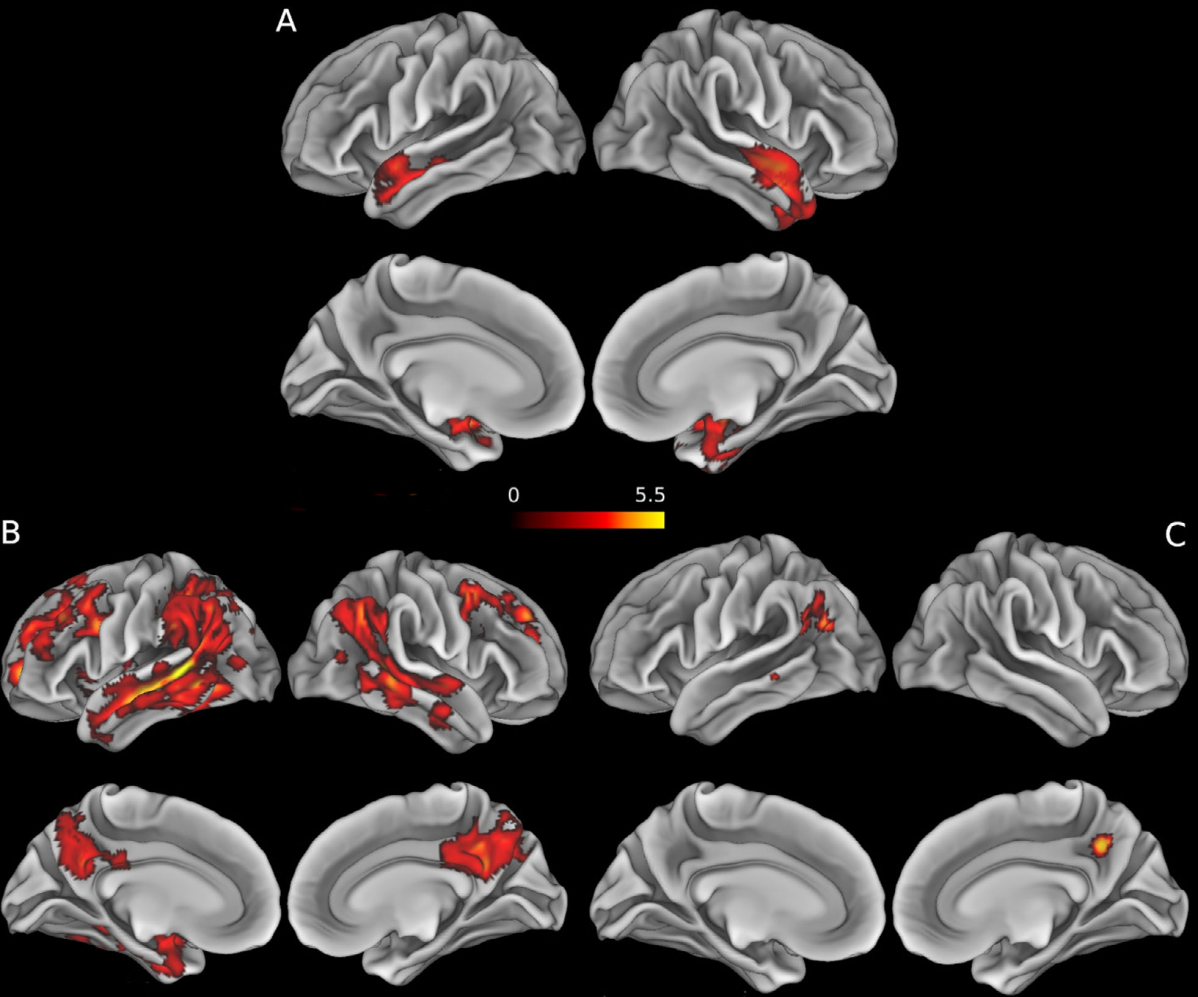
Results of the voxel‐wise analysis directly comparing patient groups. 1. Clusters display the *t*‐values for the significant results (*P* < 0.05 FWE) of the between‐group voxel‐wise analyses. Age, sex, and imaging protocol were included in every model as nuisance covariates. (A) contrast LBD+AD < LBD−AD. (B) contrast AD < LBD−AD. (C) contrast AD < LBD+AD. For visualization purposes, the results have been overlapped to an inflated mesh of the MNI space (Montreal neurological institute template) ICBM‐152 using the Connectome Workbench (v.1.3; https://github.com/Washington‐University/workbench).

The direct comparison between patient groups revealed areas of neocortical thinning in the LBD+AD group relative to the LBD−AD group in the temporal pole, parahippocampal gyrus and in the superior temporal gyrus. The clusters of significant results were bilateral, although more extensive in the right hemisphere. There were no clusters with greater cortical thinning in LBD−AD than LBD+AD. The comparison between LBD−AD and AD showed temporal, parietal, and frontal cortical thinning in AD, while no region appeared to be more atrophic in the LBD−AD group than in AD group. The contrast between AD and LBD+AD showed greater cortical thinning in AD in the angular gyrus (bilaterally), left lateral occipital sulcus, right precuneus and a small cluster in the left middle temporal gyrus. There were no clusters with greater cortical thinning in LBD+AD than AD (see Fig. 1 and Table 4).

**Table 4:** Results of MRI cortical thickness analysis using voxel‐wise comparisons directly comparing groups of patients.

LBD+AD < LBD−AD				
	*t*‐value	*x*	*y*	*z*
Region				
Right parahippocampal gyrus	**4.77**	**26**	−**10**	−**12**
Right temporal pole	4.4	48	4	−20
Right temporal fusiform cortex	4.17	28	−2	−32
Right middle temporal gyrus	4.1	56	−10	−14
Right superior temporal gyrus	4.09	56	2	−10
Left planum polare	**5.09**	−**44**	**0**	−**18**
Left superior temporal gyrus	4.17	−54	4	−10
Left temporal pole	4.13	−50	6	−10
Left parahippocampal gyrus	3.89	−28	2	−16
AD < LBD−AD				
Left middle temporal gyrus	**6.29**	−**48**	−**42**	**4**
Left angular gyrus	5.1	−46	−50	22
Left superior temporal gyrus	4.99	−48	−16	−8
Right precuneus	5.04	14	−66	40
Right middle temporal gyrus	**5**	**60**	−**38**	**0**
Right angular gyrus	4.61	48	−56	40
Lateral occipital cortex	4.57	42	−58	42
Left frontal pole	**4.93**	−**20**	**56**	**2**
Left superior frontal gyrus	4.24	−20	28	38
Left middle frontal gyrus	4.19	−34	30	36
Right frontal pole	**5.34**	**36**	**40**	**38**
Right middle frontal gyrus	4.66	42	8	48
Left middle frontal gyrus	**4.61**	−**38**	**14**	**48**
Left insular cortex	**3.66**	−**34**	−**8**	**6**
Left posterior cingulate gyrus	**3.41**	−**10**	−**24**	**36**
Right inferior frontal gyrus pars opercularis	**2.96**	**38**	**14**	**28**
AD < LBD+AD				
Left angular gyrus	**4.86**	−**44**	−**50**	**22**
Left lateral occipital cortex	4.76	−48	−66	28
Right precuneus	**5.17**	**12**	−**52**	**38**
Right angular gyrus	**6.02**	**40**	−**56**	**42**
Left middle temporal gyrus	**4.2**	−**68**	−**38**	**‐8**

Coordinates and *t*‐values are reported for the cortical voxel at the peak statistical significance for each cluster that survived FWE correction for multiple comparisons at *P* < 0.05. In bold: cluster peak, followed by sub‐peak coordinates.

### Regional MRI analysis

There was no difference in the w‐scores of the macro‐ROIs between the comparable regions of the left and right hemispheres (all *P* > 0.2). Therefore, these ROIs were averaged across hemispheres for subsequent analyses. The results showed that w‐scores differed across groups in both the lateral temporal ROI [*F*(2, 91) = 9.96, p‐FWE < 0.001] and the lateral parietal ROI [*F*(2, 91) = 7.1, p‐FWE < 0.01]. Post hoc tests showed that, in the lateral temporal ROI, both AD and LBD+AD have significant cortical thinning relative to the LBD−AD group (*P* < 0.05), while LBD+AD and AD were similar. In the parietal ROI, AD had cortical thinning relative to both LBD+AD and LBD−AD groups, while LBD−AD and LBD+AD did not differ from one another. The w‐scores in the medial temporal, medial parietal, and lateral frontal ROIs only reached a nominal significant level of *P* < 0.05 uncorrected for multiple comparisons [medial parietal: *F*(2, 91) = 3.6; MT: (2, 91) = 3.8; lateral frontal: *F*(2, 91) = 3.8]. A post hoc Tukey test to explore group differences in these regions suggested that the AD group has cortical thinning in all three ROIs compared to the LBD−AD at *P* < 0.05, while the LBD+AD did not differ significantly from either AD or the LBD−AD groups in these ROIs.

**Figure 2:**
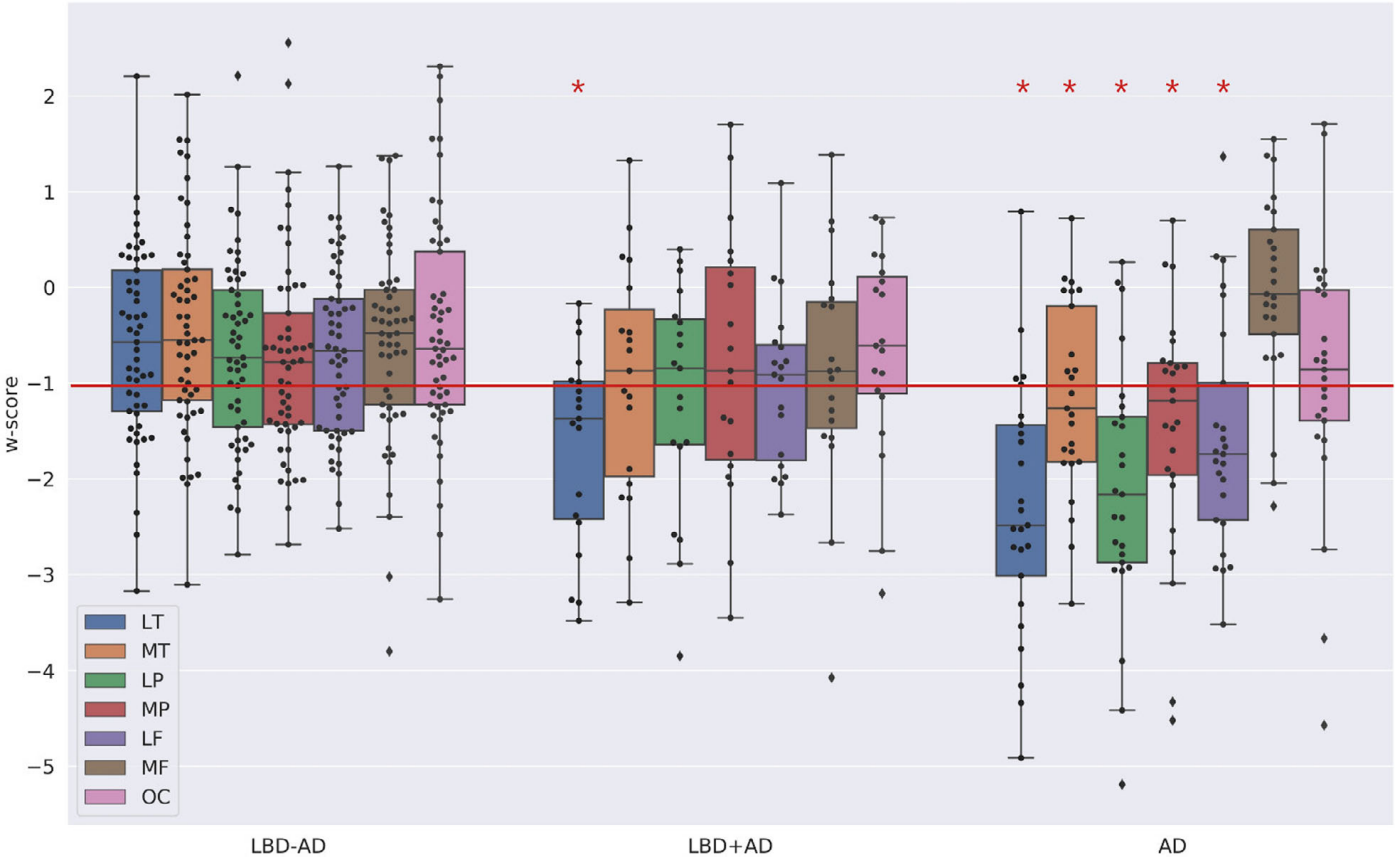
W‐score of macro‐ROIs across patients groups. Abbreviations: LT, lateral temporal ROI; MT, ventro‐medial temporal ROI; LP, lateral parietal ROI; MT, medial parietal ROI; LF, lateral frontal ROI; MF, medial frontal ROI; OC, occipital ROI. The plot represent the w‐score for each macro‐ROI in each group of patients. Each box extends from lower to upper quartile values, with line at median. Whiskers extend from box to show the range of the data. * = region in which more of the 50% of the cases lie below the threshold of w‐score = −1. Following the cut‐off of −1 (red line) for pathological thinning, it appeared that in the AD group more than 50% of the participants lay below this cut‐off in all regions except the medial frontal and the occipital ROIs while in the LBD+AD group 50% of the participants lay below the −1 cut‐off only in the lateral temporal ROI [lateral temporal vs. ventro‐medial temporal: *x* ^2^ = 4.07, *P* < 0.05; lateral temporal vs. lateral parietal: *x* ^2^ = 4.07, *P* < 0.05; lateral temporal vs. medial parietal: *x* ^2^ = 5.40, *P* < 0.05; lateral temporal vs. lateral frontal: *x* ^2^ = 5.40, *P* < 0.05; lateral temporal vs. medial frontal: x^2^ = 5.40, *P* < 0.05; lateral temporal vs. medial parietal: *x* ^2^ = 8.61, *P* < 0.01]. In no region did more of the 50% of LBD−AD participant lay below the cut‐off.

As illustrated in Figure 2, we selected a cut‐off threshold at w‐score ≤−1 to examine relative patterns of cortical thinning within each group. In the AD group, more than 50% of the participants lay below this cut‐off in all regions except the medial frontal and occipital ROIs. By comparison, analysis of the LBD+AD group showed that more than 50% of the participants lay below the −1 cut‐off only in the lateral temporal ROI, and a chi‐square test confirmed that the number of LBD+AD patients for whom the lateral temporal ROIs was below the cut‐off is significantly different from the number of LBD+AD for whom the other regions lie below the cut‐off (*P* < 0.05 for all; see Fig. 2). In the LBD−AD group, no region had more of the 50% of LBD−AD participants below this cut‐off. Taken together, the regional analyses based on w‐scores confirmed the results of the voxel‐wise comparison, showing that the LBD+AD group exhibits relative cortical thinning in the lateral temporal regions, while the AD group has relatively widespread cortical thinning and the LBD−AD group has comparatively modest cortical thinning.

### Association between cortical thinning and digital histopathology

**Figure 3:**
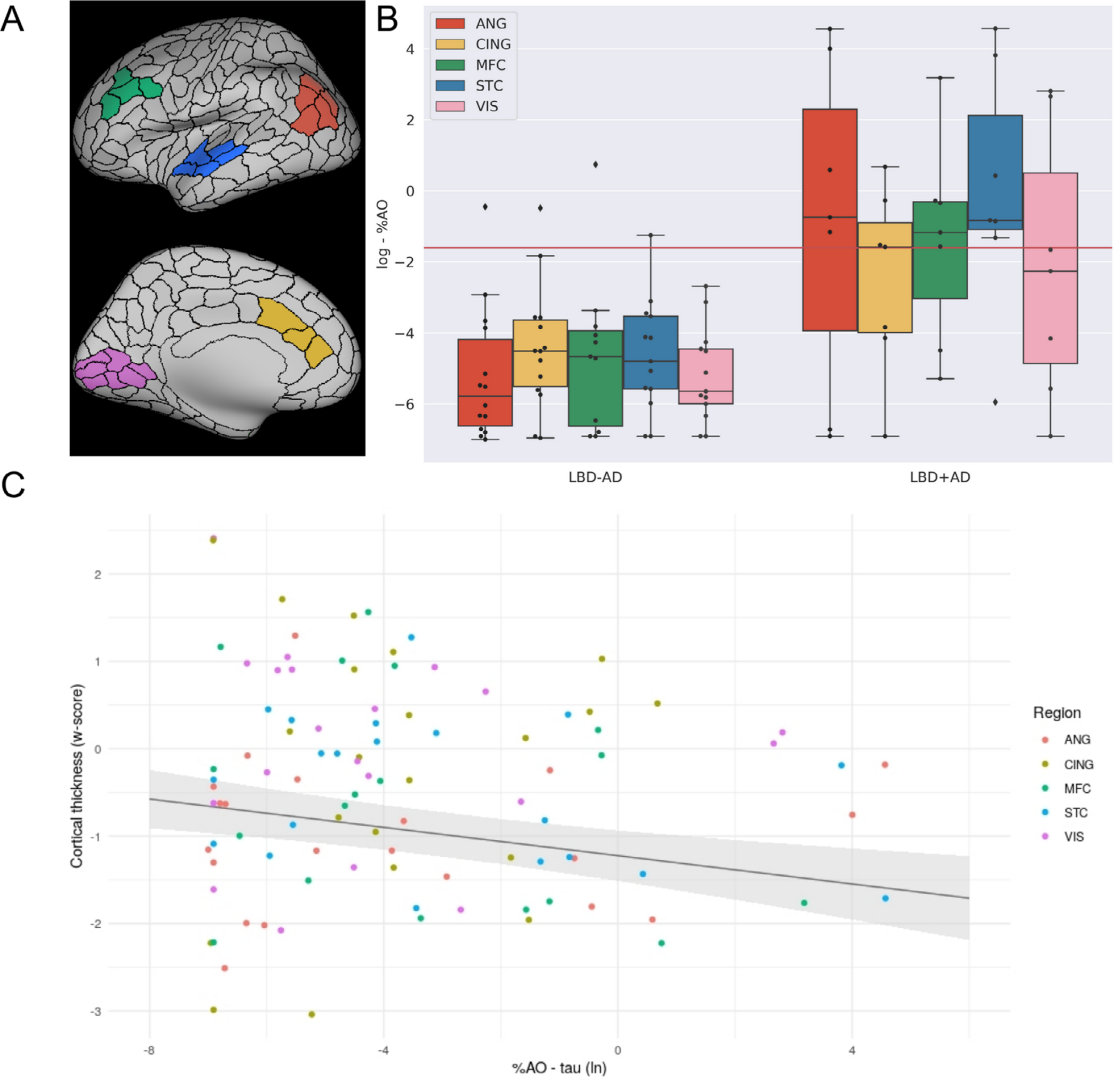
Association between the in vivo estimates of cortical thinning and tau accumulation. Abbreviations: %AO, percentage of area occupied; ANG, angular gyrus; CING, anterior cingulate cortex; MFC, medial frontal cortex; STC, superior temporal cortex; VIS, visual cortex. (A) representation of the MRI‐ROIs on an inflated brain (Green = MFC, Blue = STC, Red = ANG, Yellow = CING and Pink = VIS). (B) Box‐plots depict the ln %AO distribution of tau pathology across the sampled regions. (C) The scatter plot represents the w‐score of median cortical thickness values plotted as a function of the percentage of area occupied of reactivity for tau in the tissue sample (log transformed). The regression line represents the fixed effect of the %AO and the gray area around the regression line covers the standard error around the regression estimate.

To relate ante‐mortem imaging to neuropathology, we examined the relationship between MRI cortical thickness and neuropathological burden of tau, A*β*, and SYN % area occupied in the autopsied LBD. A mixed effects model revealed a significant inverse association between antemortem estimates of cortical thickness, and post‐mortem %AO for tau in the tissue sample from the corresponding region [ln‐%AO: *β* = −0.12, SE = 0.06, *t*‐value = −2.13, *P* < 0.05] (Fig. 3, panels A–C). The analyses relating cortical thinning to both %AO for A*β* and %AO for SYN revealed no significant association [A*β* ln‐%AO: *β* = −0.02, SE = 0.06, *t*‐value = −0.45, *P* > 0.6; SYN ln‐%AO: *β* = −0.13, SE = 0.07, *t*‐value = −1.99, *P* = 0.06] (see Table 5 for the complete statistical results). The regional distribution of tau, A*β*, and SYN is summarized in Table 6. As expected, there was greater tau and A*β* %AO in these neocortical regions in LBD+AD versus LBD−AD [average cortical ln‐%AO for tau: *β* = −3.6, SE = 0.95, *t*‐value = −3.7, *P* < 0.01; average cortical ln‐%AO for A*β*: *β* = −4.2, SE = 1.3, *t*‐value = −3.2, *P* < 0.01]. There was also a greater average neocortical SYN %AO in LBD+AD than LBD−AD [*β* = −3.4, SE = 0.6, *t*‐value = −5.8, *P* < 0.001]. Figure 3 (panels A–B) shows the burden of tau pathology in each region for each participant (ln‐%AO). We observed the greatest tau accumulation in the superior temporal ROIs in the LBD+AD group. In order to compare pathologic tau burden across regions, we used a threshold to define significant tau pathology derived from mean superior temporal cortex ln‐%AO measurements of samples rated with intermediate‐to‐high ordinal ratings (threshold = −1.6). We found that six out of seven LBD+AD cases are above this cut‐off (i.e., greater intermediate‐to‐high tau pathology) while in the less affected primary visual cortex ROI, the same threshold was surpassed only in two LBD+AD participants [STC vs. VIS: *x* ^2^ = 4.7, *P* < 0.05].

**Table 5:** Results of the association between in vivo measures of cortical thickness and post‐mortem measure of protein accumulation.

Cortical thickness ~ tau			
	*β*	Standard error	*t*‐value
Fixed effect			
%AO	−0.12	0.06	−2.13^1^
Age at scan	0.06	0.02	3.42
Sex	−0.20	0.56	−0.35
Imaging protocol^2^	−0.24	0.29	−0.81
%AO * MRI‐death interval	0.002	0.002	1.43
Region: CING	0.76	0.32	2.35
Region: MFC	0.37	0.34	1.11
Region: STC	0.58	0.33	1.78
Region: VIS	0.99	0.33	3.03
Cortical thickness ~ A*β*			
%AO	−0.02	0.06	−0.45
Age at scan	0.06	0.02	3.34
Sex	−0.07	0.58	−0.11
Imaging protocol^2^	−0.05	0.28	−0.18
%AO * MRI‐death interval	0.0002	0.003	0.09
Region: CING	0.72	0.33	2.17
Region: MFC	0.38	0.34	1.14
Region: STC	0.47	0.33	1.42
Region: VIS	0.89	0.34	2.61
Cortical thickness ~ alpha‐Synuclein			
%AO	−0.13	0.07	−1.99
Age at scan	0.06	0.02	3.28
Sex	−0.10	0.57	−0.17
Imaging protocol^2^	−0.12	0.27	0.45
%AO * MRI‐death interval	0.005	0.002	2.22
Region: CING	0.84	0.34	2.48
Region: MFC	0.39	0.32	1.23
Region: STC	0.51	0.32	1.61
Region: VIS	0.98	0.34	2.89

Abbreviations: %AO, percentage of area occupied for tau A*β* or alpha‐synuclein; ANG, angular gyrus (reference region in the model as previously reported^2^); CING, anterior cingulate cortex; MFC, medial frontal cortex; STC, superior temporal cortex; VIS, visual cortex for tau, A*β* or alpha‐synuclein.

^1^ Significance at *P* < 0.05 using the Kenward–Roger approach.

^2^ This subset of participants have been scanned with only two of the four imaging protocols.

**Table 6:** Regional distribution of pathology expressed percentage area occupied (%AO).

Pathology	Region	LBD−AD, *N* = 14	LBD+AD, *N* = 7
Tau	ANG	0.05 (0.17)	21.88 (38.35)
	CING	0.07 (0.16)	0.46 (0.72)
	MFC	0.20 (0.63)^3^	3.73 (8.99)
	STC	0.03 (0.08)^1^	20.68 (37.42)
	VIS	0.01 (0.02)^1^	4.46 (7.54)
A*β*	ANG	1.99 (2.84)^1^	7.07 (4.13)
	CING	2.04 (4.21)	8.49 (4.22)
	MFC	2.24 (3.25)	8.10 (4.27)^4^
	STC	1.22 (1.72)	4.48 (3.28)
	VIS	0.69 (1.18)^2^	3.28 (1.78)
SYN	ANG	0.32 (0.71)	15.00 (20.30)
	CING	7.20 (13.67)	29.66 (17.67)
	MFC	1.28 (2.57)	19.45 (32.42)
	STC	0.56 (0.85)	23.55 (32.44)
	VIS	0.03 (0.05)	0.73 (1.44)

Abbreviations: ANG, angular gyrus; CING, anterior cingulate cortex; MFC, medial frontal cortex; STC, superior temporal cortex; VIS, visual cortex. The %AO is reported as mean (standard deviation). N, number of slices for each region.

^1^ 13 slides available.

^2^ 12 slides available.

^3^ 11 slides available.

^4^ 6 slides available.

## Discussion

This study examined the association between MRI measures of cortical thinning and neuropathology in biologically defined LBD subgroups stratified by the presence or absence of AD co‐pathology. The voxel‐wise MRI analysis showed more prominent neocortical thinning in temporal neocortical regions in the LBD+AD group relative to the LBD−AD group, which was confirmed by regional analyses. Comparisons with AD showed that cortical thinning in LBD+AD is relatively specific to the lateral temporal lobe, while cortical thinning in an AD reference group appeared to encompass temporal, parietal, and frontal cortical regions. To examine the pathological basis for MRI cortical thinning, we directly compared ante‐mortem cortical thinning with parametric digital measures of neuropathologic burden. This analysis revealed a significant inverse correlation between ante‐mortem estimate of cortical thickness and tau neuropathologic aggregation across the sampled regions. However, A*β* and SYN were not significantly associated with cortical thinning. These results are consistent with the hypothesis that LBD pathophysiology is impacted by AD co‐pathology, and particularly that there is a strong link between tau pathology and in vivo evidence of neurodegeneration in LBD. This observation supports the clinical utility of subcategorizing LBD in terms of the presence or absence of AD co‐pathology.

Only few studies have previously investigated the impact of pathology on in vivo imaging markers of disease in LBD. van der Zande and colleagues^15^ found that DLB patients with AD co‐pathology (based on CSF markers) have greater medial temporal lobe atrophy. Moreover, smaller hippocampal and amygdala volumes associated with higher Braak neurofibrillary tangle stage in DLB has been reported by Kantarci and colleagues.^4^ Nedelska and colleagues^16^ reported a temporo‐parietal pattern of increased atrophy over two time points in DLB with neuropathological evidence of AD co‐pathology relative to DLB without AD co‐pathology. While some of the results of previous studies differ from our findings, methodological aspects challenge direct comparisons between these reports. The results of van der Zande et al are based on visual rating of atrophy, while the results of Nedelska and colleagues included data acquired with 1.5T scanners and without digital histological evaluation of regional pathologic burden. The present study instead quantified cortical thinning from high‐resolution T1‐weighted sequences acquired at 3T and related these observations directly to parametric measures of tau, A*β*, and SYN pathology post‐mortem. Moreover, our focus on cortical regions does not allow a direct comparison with studies that reported volumetric difference in hippocampus and amygdala, although the voxel‐wise analysis suggested cortical thinning in the LBD+AD relative to the LBD−AD in anterior and medial temporal areas neighboring the hippocampus, namely the parahippocampal gyrus. Our cohort also differed from these studies, focused on DLB, and uniquely encompassed the full range of LBD clinical diagnoses from movement disorder and cognitive clinical research cores rather than focusing on a specific LBD clinical syndrome, and this broader perspective is helpful to study underlying biology of LBD.^42^

Increasing evidence is revealing the impact of AD co‐pathology on LBD pathophysiology. For example, AD co‐pathology appears to influence the onset of dementia in LBD,^5^, ^43^, ^44^ underlining the association of AD co‐pathology with older age, shortened motor‐cognitive deficits interval, and decreased survival in PDD.^45^, ^46^, ^47^ In neuropathological studies, tau neurofibrillary tangle pathology in LBD is most often shown to have a distribution similar to typical AD using conventional neuropathological staging methods.^47^ However, the development of more granular, digital histological measurements has revealed potential avenues of improvement over traditional methods that can further elucidate the relative roles of tau and A*β* pathology in LBD. Typical neuropathological examinations tend to reflect the distribution of pathology with less emphasis on the severity of regional pathologic burden. Our objective, continuous, digital metrics facilitated regression analyses with ante‐mortem imaging that otherwise would not be possible using traditional ordinal pathology rating scales^17^, ^29^, ^48^. Recent studies employing this digital technique have suggested reduced tau pathology in the medial temporal lobe^17^ and greater relative tau pathologic burden in temporal neocortex in LBD versus AD despite comparable Braak stage across groups.^18^ Here, we replicated our previous digital pathologic data^18^ in a largely non‐overlapping dataset using open‐source digital image analysis tools and found greater relative tau neuropathology in the STC compared to other regions in LBD+AD, while A*β* regional severity was more diffuse (Table 6) emphasizing the regional specificity of tau pathology and resultant neurodegeneration in LBD.

Another line of evidence elucidating the role of AD pathology in LBD comes from positron emission tomography (PET) studies. Tau‐PET uptake, most often quantified by ^18^F‐flortaucipir, has been shown to be elevated in some LBD patients, especially in DLB who may be more likely to bear tau and A*β* neuropathology, although the degree of uptake is typically less than that observed in AD.^19^, ^20^ Moreover, the patterns of regional tau‐PET uptake in LBD have differed from that seen in AD, suggesting an atypical pattern of spreading of tau pathology with relatively preferential temporoparietal distribution. Similar to previous PET data, we also found cortical thinning to be less widespread in LBD+AD versus AD (Fig. 1). Considering the cross‐sectional nature of the present study, it is not possible to determine how cortical thinning patterns may change over time in LBD. Moreover, PET studies could have captured temporoparietal accumulation of tau that could antedate neurodegeneration detected by structural MRI.

The close association between tau pathologic burden and measures of neurodegeneration such as MRI cortical thinning is well recognized in AD research^8^, ^49^ and reinforced also by recent in vivo tau‐PET evidence.^50^, ^51^ While the exact biological mechanism and toxic species of tau leading to cortical thinning is unclear (i.e., fibrils, oligomers, tangles, neuronal death),^50^ our data here in LBD patients align with the AD literature linking tau neurofibrillary pathology closely with neurodegeneration.^8^ However, future large‐scale autopsy work is needed to fully compare mechanisms of tau‐mediated degeneration in LBD and AD.^52^ Such a link between tau and markers of neurodegeneration does not necessarily undermine the central role played by SYN in LBD; SYN pathology may be more closely related to synaptic dysfunction than neuronal death^5^ and subsequent atrophy detected on in vivo structural imaging. Moreover, the detrimental effect of SYN misfolding could be more critical in early stages of the disease course, for example, while the analysis reported in the present study is based on MRI scans obtained later in the course of disease after the onset of cognitive impairments. Indeed, we found a trend that approached statistical significance for an association between ante‐mortem cortical thinning and SYN burden (Table 5). The interaction between SYN and AD co‐pathology in LBD is likely to be complex. For example, models of SYN propagation have shown that SYN can interact with both tau and A*β* to promote misfolding and accumulation of pathology.^53^, ^54^ Recent studies have also found two distinct strains of SYN with differential abilities to cross‐seed and promote the fibrillization of tau.^55^ It is intriguing to speculate that different strains of SYN, or other factors such as genetic risk, could be partially responsible for the different degree of tau pathology and, in turn, for the phenotypical heterogeneity, including heterogeneity in neurodegeneration observed on structural MRI that characterizes LBD. In the context of clinical LBD, current clinical criteria note the absence of appreciable cortical atrophy as supportive evidence for DLB. The present results instead support the potential use of cortical thickness measures in the temporal lobe for helping the differential diagnosis between LBD−AD and LBD+AD biological groups, especially in absence of other AD‐specific biofluid and PET scan biomarkers. However, further studies investigating sensitivity and specificity of the pattern of cortical atrophy at the single subject level are necessary to test the reliability of a potential marker based on cortical thickness measures.

The strengths of our study include the multi‐modal approach, the use of quantitative imaging analyses, and our novel, validated, parametric analyses of digitized histopathology of specific pathologic proteins. However, important limitations should be considered when evaluating the present study. While post‐mortem data are very rare in patients with ante‐mortem MRI, our cohort is relatively small (*N* = 21) and future multi‐center studies will be needed to replicate our results. The division between LBD+AD and LBD−AD was based on rigorous neuropathological criteria in 21 participants, but we employed an autopsy‐based CSF cut‐off for this discrimination in the remaining cases. While we used an autopsy‐validated cutpoint to establish AD co‐pathology in LBD, there is a possibility that there was a subset of non‐autopsied LBD patients without underlying alpha‐synucleinopathy. Indeed, clinical diagnosis of PD/PDD is less specific in early stages of disease^56^; however, most of our PD patients were scanned at the time of cognitive impairment several years into the disease (Tables 1 and 2) where a misdiagnosis of underlying pathology is much lower. Thus, our patients were carefully clinically characterized to reduce possible misdiagnosis in non‐autopsied LBD. A related limitation is the lack of neuropathological confirmation in the cohort meeting rigorous clinical diagnosis of AD in our AD reference group. While AD CSF biomarkers are more established in AD,^8^ a portion of our AD reference group could harbor some degree of alpha‐synuclein,^57^, ^58^ TDP‐43,^59^, ^60^ or other age‐related co‐pathologies.^61^, ^62^ These pathologies are largely focused in the medial temporal lobe in AD^2^, ^59^ while our focus was largely on cortical thinning in the neocortex; however, we cannot entirely rule out a possible contribution from these and other age‐associated co‐pathologies to the more wide‐spread cortical thinning we observed in our AD reference group compared to our LBD patient groups of interest. Finally, participants have been screened for significant cerebrovascular disease at recruitment and while we report rare autopsy‐confirmed imaging data with a unique strategy of autopsy‐validated biomarkers to stratify our LBD in biologically based groups, these limitations highlight the importance of brain donation and autopsy‐confirmation in neurodegenerative disease research. Another limitation is the inevitable time interval between the MRI scan and neuropathological examination. We attempted to control this in two ways: (1) by including the interval as a covariate in all analyses and (2) selecting only cases with an MRI scan within 5 years from death. While we performed a unique analysis directly relating MRI cortical thinning to regional histopathology, another limitation is the potentially limited spatial overlap between MRI‐ROIs and tissue samples due to the inherent difference in scale between measurements. Moreover, while the digital pathological assessment included three different pathologies across five standardized regions in 21 cases (311 slides total), neuropathological sampling was relatively sparse compared to whole‐brain MRI data. Therefore, the present findings should be replicated in future studies with high‐density neuropathological sampling procedures.

With these caveats in mind, the present study provides evidence that LBD patients with significant AD co‐pathology are characterized by a pattern of cortical MRI thinning that diverges from both LBD patients with a relative pure alpha‐synucleinopathy and from typical AD patients. Moreover, we observed a specific association between post‐mortem tau pathologic burden and in vivo measures of cortical thinning, suggesting that tau pathology specifically contributes to phenotypic heterogeneity in LBD. These results support the use of in vivo AD biomarkers in the subcategorization of LBD patients based on biological evidence of the underlying pathology.

## Conflicts of Interest

The authors report no competing interests.

## Author Contributions

NS, DGC, DW, JQT, ACP, CTM, DW, MG, DJI: conception and design of the study; NS, DGC, CAO, SNV, LMS, ND, JFM, JED, AFD, MAS, EBL: acquisition and analysis of data; NS, DGC, CAO, DW, MG, DJI: drafting a significant portion of the manuscript or figures.
